# Climate change and health in school-based education: A scoping review protocol

**DOI:** 10.1371/journal.pone.0282431

**Published:** 2023-03-01

**Authors:** Lira Ramadani, Sudeepa Khanal, Melanie Boeckmann

**Affiliations:** 1 Faculty of Health Sciences, Universitätsstraße Bielefeld, Bielefeld, Germany; 2 University of Bremen, Faculty of Human and Health Sciences, Mary-Somerville, Bremen, Germany; Hacettepe University: Hacettepe Universitesi, TURKEY

## Abstract

Taking into account the adverse impacts of climate change on human health, the importance of increasing knowledge and gaining essential skills is necessary to mitigate and adapt to its impacts and protect human health. Researchers and experts are urging for more research in the climate-health nexus, as well as calling for efforts that establish climate and health educational goals. They encourage the development of agreed upon, articulated science-based curricula and resources addressing climate-health issues. This review aims to map out the current state of integration of climate change education in school-based education across the world and identify the human health topics included. Furthermore, it aims to explore the extents to which levels of prevention and health co-benefits of climate mitigation and adaptation are covered within the framework of school-based climate change education. Five electronic databases will be searched for peer reviewed articles in English, from year 2000-to May 2022. The findings from the study will be useful to school curricula developers looking to expand climate change education. This review will also highlight potential research gaps in education on climate change-related health in schools. The scoping review was preregistered with the Open Science Framework [registration DOI: https://doi.org/10.17605/OSF.IO/8U5GK].

## 1. Introduction

The levels of greenhouse gases have been increasing since the early industrial revolution, mainly due to manmade activities such as growing burning of fossil fuels along with increases in deforestation, animal husbandry, irrigated agriculture and cement manufacture [1]. Climate change is causing temperature increase, hydrologic cycle changes, sea level rises, more frequent extreme weather events and heat events [2]. These weather and climatic changes will consequently impact the incidence and geographic spread of climate-sensitive vector-, food-, and waterborne diseases, and increase diseases associated with air pollution and aeroallergens [3, 4].

Poor air quality and hot weather due to climate change are becoming a major public health concern as there is an increasing excess of morbidity and mortality cases related to them [1, 5]. WHO estimated that modifiable environmental factors are responsible for 23% of deaths across the world and 26% of deaths of children under the age of 5 [6]. Climate change is increasing the burden of climate-sensitive health determinants and outcomes for the global population, with the most vulnerable groups being children, the elderly, people with preexisting health conditions, outdoor workers, people of color, and people with low income. Children are at a higher risk due to their potentially greater exposures to climate-induced health risks, greater sensitivity, their dependence on caregivers [7, 8], physical, physiologic, and cognitive immaturity [9, 10].

Acknowledging the impact of a drastically changing climate, its effects on earth as well as concern for its anthropological causes, the United Nations Framework Convention on Climate Change [UNFCCC] introduced nearly three decades ago, [11] recognizes education as a crucial way to address the challenges of climate change on an international scale [11, 12]—as such, the UNFCCC lays the foundation for climate change education as it is seen today [13]. In today’s general consensus, climate change is no longer seen simply as merely a scientific phenomenon but regarded as “a complex climate socio-scientific issue” [14] [p. 68].

Early child development and education has been proven to be pivotal, not only to health in childhood and later life of an individual, but also impactful on a societal level [15]. Early education possibly ameliorates issues of health inequities, through enabling protection against adverse exposures during childhood thereby potentially reducing health inequity in adulthood [15]. Given the strong link between education and the health of younger generations [16], schools play a vital role, especially primary education, as they reach children at a development stage. At this age-period, children start expressing behavior and attitude, which may be potentially impactful for their future health [15, 17]. To this end, schools are a convenient location to offer health education, either embedded in scientific subjects, physical education or as specific health courses [18].

Both mitigation and adaptation are essential in minimizing the impact of climate change on health [5]. Mitigating the climate change impacts can have direct and immediate health benefits whereas adaptation is necessary as it will decrease and avoid diseases, health-care expenditure and lost productivity [2]. Hence, addressing climate change would be highly advantageous pertaining to global health by reducing emissions and cooling temperatures through changes in the built environment [5, 19]. Climate change education combines the scientific basics of climate change with a broader view on the interconnectedness between climate and multiple parts of human societies [20] and equips its learners with both combined mitigation and adaptation capabilities [3, 14, 21]. In doing so, climate change education aims to enable people across societies to make informed decisions; incite attitude and behavior changes [22, 23] and gain essential skills to mitigate and adapt to climate change [10, 20, 21] and thus equip them with “climate literacy” [20, 24].

Taking into account the adverse effects of climate impacts on health, there are emerging attempts to build momentum to shift “climate literacy” to “climate and health literacy” as recent research is increasingly emphasizing the need that health professionals, governments, businesses and the general public proactively address both climate change and its implication for public health [19, 25–27]. However, existing educational frameworks still fail to adequately prepare students, are limited to health professionals and include limited content coverage and perspective [27]. A broader educational agenda at all levels of education system as a crucial component of the global response to climate change is proposed [26–28] with schools being the most common setting identified in existing literature for the promotion of climate and health education [29–31]. Limaye et al. [2020] proposes a definition for climate and health literacy by adapting the US climate literacy principles. They define a climate and health literate individual as one that “can recognize direct and indirect linkages between climate change and health, communicate risks, assess data, comprehend uncertainty, and make informed and responsible personal decisions or advocate for broader policies that protect health” [27] [p. 6]. They state that proficiency in climate and health literacy is anticipated to be developed over time, with younger students obtaining functional levels during primary and secondary education [27].

Evidence and work on the effectiveness of climate change education is still lacking, [32] however research in this nexus is increasingly gaining traction [3, 30]. A report found that the majority of climate change programs address the causes of climate change but fewer tend to focus on issues regarding climate change mitigation, adaptation and impact reduction [30]. This study however did not address the health linkages covered in climate change education. Osama et al. [33] scoping review protocol aims at exploring the linkages of climate change and health offered in online platforms. However, the results of this scoping review have still not been published and are not available [33].

This scoping review intends to fill the gap in the research on the links between climate change education and health, with a specific focus on school-based learning environments, considering their vital role in impacting attitudes and behavior [15, 17]. Specifically, it aims to explore the extent to which the levels of prevention, climate mitigation and adaptation, and health co-benefits are covered in school-based climate change education.

## 2. Materials and methods

The scoping review protocol will be reported in accordance with PRISMA Extension for Scoping Reviews [PRISMA-ScR]: Checklist and Explanation [34]. The review will be carried out in accordance with this protocol, and details of any changes to this protocol will be reported in the final manuscript. Additionally, the development and planning of the review and protocol is guided by the JBI [The Joanna Briggs Institute] approach and the PCC [Population [or participants]/Concept/Context] framework. The JBI approach will be used for the planning and completion of this review, being the most updated and rigorous approach to date for scoping reviews [35].

A scoping review was concluded to be the most appropriate to address the research aims as it provides an overview of the volume and distribution of the evidence base as well as highlights where more research is warranted. Scoping reviews serve to synthesize evidence and assess the scope of literature on a topic. Among other objectives, scoping reviews help determine whether a systematic review of the literature is warranted [34]. The scoping review was preregistered with the Open Science Framework [registration DOI: https://doi.org/10.17605/OSF.IO/8U5GK]. Currently, the data extraction from the included studies is being conducted.

### 2.1. Identifying the review question

Research questions were developed to address the research aims: “What health topics are addressed in climate change education programs offered in schools?” and “Are levels of prevention, health co-benefits of climate mitigation and adaptation covered?”.

Research question formulation was guided by item 4 in the PRISMA scoping review extension checklist [34]. Through the PCC framework, the main elements were identified (Population or participants/Concept/Context), which helped to conceptualize the review questions and the study objectives.

PCC (Population/Concept/Context) [36] framework is recommended by JBI to identify the main concepts in the primary review questions. The framework also informs the search strategy. S1 Appendix includes the PCC framework along with the keywords and medical headings used.

### 2.2. Identifying relevant studies

A review of the literature will be performed in PubMED, EMBASE, Web of Science [Core Collection], GreenFILE, and Education Resource Information Centre. Peer-reviewed articles will be included. Grey literature databases and repositories will not be explored.

PubMed searches were conducted initially to identify appropriate keywords and MeSH terms and develop the search strings. Then fitting search strings were developed for the other databases as well. The search sting includes MeSH and keywords related to “education”, “climate change”, “health”, “schools”. Truncation (‘*’), wildcards, and Boolean operators (AND/OR) are used, as appropriate, to form the search terms.

The librarian of the University of Bielefeld was consulted for the development of the search strings for all six databases. The search strings for each database can be found in S2 Appendix.

### 2.3. Study selection

The study will consider all relevant studies from 2000 onwards without any geographical restriction. The review will focus on primary and secondary, thus excluding higher education. There has been limited research throughout the 1990s regarding climate change education, with the early 2000s seeing emerging research in this field [29]. Additionally, research on the climate change and health nexus gained traction after the Millennium Development Goals adoption in 2000 [37].

Only publications in English language will be considered. Grey literature available in the public domain from websites, in the form of curricula, journal, reports, process documentation etc., will not be considered for the review, due to the methodological challenges that it presents and the reduced reproducibility.

#### 2.3.1. Exclusion criteria

- Publications published prior to 2000
- Non-English publications
- Grey literature, review papers, book reviews, unpublished conference papers, books and chapters, commentaries and editorials

Titles and abstracts yielded by the search strategy will be uploaded to Endnote, where articles from different databases will be combined and duplicates will be removed. Two reviewers (LR, SK) will independently screen all titles and abstracts against the eligibility criteria to identify relevant studies. Studies that meet the criteria will then be accessed as full-text and screened against the eligibility criteria. A log of the excluded studies will be kept stating the reason for exclusion. In cases of ambiguity or disagreement, a decision of eligibility for inclusion will be made after consultation with a third reviewer (MB). Further, any study found not matching the inclusion criteria will be removed during the full-text review.

### 2.4. Extracting data

LR will perform data charting in Microsoft Excel. The first version of data extraction sheets will be piloted by LR and SK and adjusted if necessary. LR will mainly perform data extraction for included references. SK and MB will countercheck the work of LR.

### 2.5. Collating, summarizing and reporting the results

Data will be extracted and collected using a custom Microsoft Excel spreadsheet. Extracted data will include authors’ name and publication year, type of publication, age group of learners, geographical location, educational setting, duration of program, health topics, and content focus. Finally, results will be presented in table format and summarized in text format. Finally, a narrative summary of the review findings will be provided, using frequencies and thematic analysis [38].

### 2.6. Institutional review board statement

Not Applicable. This study will be a review of already published and publicly available data retrieved from the databases mention above (see section 2.2). Ethical concerns related to the involvement of humans or other are not applicable in this case. Upon completion of the review, a manuscript for publication in a peer-reviewed journal will be prepared.

## 3. Limitations

It is possible that our review will not include all articles which have been published in every journal as some may not be accessible or may be missed due to diverse terminology used. Another limitation will be the inclusion of English language studies and literature only, as this will mean that evidence published in languages other than English will not be included in the scoping review. Also, methodology quality appraisals of the studies and evidence included in the scoping review will not be executed, which is a potential limitation. However, Arksey and O’Malley [39] and Pollock et al.[40] state that quality appraisals of evidence is not the focus of scoping reviews.

## 4. Conclusions

This review is the most recent to comprehensively report the breadth of literature on the relationship between climate change and health education in school-based education. Its main aim is to understand to what extent health topics are covered in climate change education in primary and secondary schools and the nature thereof. The scoping review will conform to the rigorous methodology manual by the Joanna Briggs Institute. The findings from this scoping review will be useful to school curricula developers looking to expand climate change education. This review will also highlight potential research gaps in education on climate change-related health in schools.

## Supporting information

S1 Checklist PRISMA-P 2015 checklist. (PDF)

S1 Appendix Appendix A—PCC framework. (PDF)

S2 Appendix Appendix B—Search strings and search run. (PDF)
