# Supplementary material for: Climate change and health in school-based education: A scoping review protocol
Source: PLoS One. 2023 Mar 1;18(3):e0282431. doi: 10.1371/journal.pone.0282431 (PMC9977044; doi:10.1371/journal.pone.0282431)
Supplement: S1 Appendix — (PDF) [file pone.0282431.s002.pdf]

Appendix A- PCC Framework (incl. alternate keywords and subject headings)

	Main Concept	Alternate keywords	Subject headings
**Participants**	- Young learners of any background, including all genders, ethnicities, races, ages		
**Concept**	-Health topics integrated in Climate Change Education<br>-Nature of the health topics<br>-Levels of prevention, health co-benefits of climate mitigation and adaptation	Climate change education, environmental education, education for sustainable development, education for sustainability<br><br>AND<br><br>Health, health promotion	Climate change, education, health
**Context**	–School-based education (Primary Schools, Secondary Schools, High Schools)<br>–No geographical restriction	Schools, school-based	schools
