# Supplementary material for: Climate change and health in school-based education: A scoping review protocol
Source: PLoS One. 2023 Mar 1;18(3):e0282431. doi: 10.1371/journal.pone.0282431 (PMC9977044; doi:10.1371/journal.pone.0282431)
Supplement: S2 Appendix — (PDF) [file pone.0282431.s003.pdf]

Appendix B - Search strings and search run (21 May 2022)

PubMed **Results:214**

Search: ((climate change education) OR ("education for sustainable development" OR "education for sustainability" OR "environmental education")) AND ("schools" OR "school-based") AND (health) Filters: from 2000 – 2022, Language: English

EMBASE **Results:168**

('climate change education' OR 'environmental education' OR 'education for sustainable development' OR 'education for sustainability') AND ('school'/exp OR school) AND ('health'/exp OR health) AND [2000-2022]/py

ERIC **Results:108**

abstract:("climate change education" OR "environmental education" OR "education for sustainable development" OR "education for sustainability") AND ("school" OR "school-based") AND "health" pubyearmin:2000 pubyearmax:2022

GreenFILE **Results:146**

(Climate change education OR environmental education OR education for sustainable development OR education for sustainability) AND school AND health
Publication Date: 20000101-20221231

Web of Science **Results: 337**

"climate change education" OR "environmental education" OR "education for sustainable development" OR "education for sustainability" (All Fields) and school (All Fields) and health (All Fields)
Timespan: 2000-01-01 to 2022-12-31 (Publication Date)
